# Measuring perceived effects of drinking an extract of basidiomycetes *Agaricus blazei *murill: a survey of Japanese consumers with cancer

**DOI:** 10.1186/1472-6882-7-32

**Published:** 2007-10-29

**Authors:** James A Talcott, Jack A Clark, Insu P Lee

**Affiliations:** 1 Center for Outcomes Research, Massachusetts General Hospital Cancer Center and Harvard Medical School, Boston, MA, USA; 2 Center for Health Quality, Outcomes, and Economic Research, Edith Nourse Rogers Memorial Veterans Hospital, Bedford, MA, and Boston University School of Public Health, Boston, MA, USA; 3 Visiting Professor, Complimentary and Alternative Medicine, Graduate Faculty of Medical Sciences, Kanazawa University, Kanazawa, Japan

## Abstract

**Background:**

To survey cancer patients who consume an extract of the Basidiomycetes *Agaricus blazei *Murill mushroom (Sen-Sei-Ro) to measure their self-assessment of its effects and to develop an instrument for use in future randomized trials.

**Methods:**

We designed, translated and mailed a survey to 2,346 Japanese consumers of Sen-Sei-Ro self-designated as cancer patients. The survey assessed consumer demographics, cancer history, Sen-Sei-Ro consumption, and its perceived effects. We performed exploratory psychometric analyses to identify distinct, multi-item scales that could summarize perceptions of effects.

**Results:**

We received completed questionnaires from 782 (33%) of the sampled Sen-Sei-Ro consumers with a cancer history. Respondents represented a broad range of cancer patients familiar with Sen-Sei-Ro. Nearly all had begun consumption after their cancer diagnosis. These consumers expressed consistently positive views, though not extremely so, with more benefit reported for more abstract benefits such as emotional and physical well-being than relief of specific symptoms. We identified two conceptually and empirically distinct and internally consistent summary scales measuring Sen-Sei-Ro consumers' perceptions of its effects, Relief of Symptoms and Functional Well-being (Cronbach's alpha: Relief of Symptoms, α = .74; Functional Well-Being, α = .91).

**Conclusion:**

Respondents to our survey of Sen-Sei-Ro consumers with cancer reported favorable perceived effects from its use. Our instrument, when further validated, may be a useful outcome in trials assessing this and other complementary and alternative medicine (CAM) substances in cancer patients.

## Background

Complementary and alternative medicine (CAM) is widely used by patients who are undergoing or have completed medical treatment for cancer [1,2]. While conventional therapies, including surgery, radiation therapies and anticancer drugs, are targeted at tumors, their side effects on normal organs may significantly compromise quality of life during and after treatment. For many patients, CAM approaches may be pursued in order to augment conventional modalities' anti-cancer effects, as well as to reduce treatment-related symptoms and other side effects that diminish their quality of life [3-5]. While the distinction between biological end points and the broader perspective of outcomes research is important to any assessment of oncology practice [6], it is particularly important when patients use CAM therapy for these less clearly defined objectives. However, few investigations have attempted to measure rigorously the goals patients have in using these substances and whether they achieve them. We have learned that measuring quality of life outcomes in cancer patients in ways that accurately reflect their perspectives is challenging [7-9]. We found an opportunity to address this challenge when asked to design a survey of Japanese users of a widely used CAM substance, Sen-Sei-Ro.

Sen-Sei-Ro is an extract derived from mushroom, Basidiomycetes *Agaricus blazei *Murill, that has been reported to have a stable antioxidant activity [10], as well as antimutagenic [11,12], antitumorigenic [13-16], chemopreventive [17] and immunostimulatory effects [17,18], and improved quality of life associated with immunological effects in a cohort of cancer patients [19]. In addition, a study of the 12,465 residents of Nagano Prefecture of Japan found lower cancer death rates among mushroom farmers compared to other residents of the prefecture [17]. Sen-Sei-Ro has been manufactured and marketed in Japan by Kyowa-S.S.I. since 1991 and, according to company estimates, has been purchased by as many as 700,000 cancer patients. In previous surveys of its consumers the company has found that improved quality of life was an important rationale for Sen-Sei-Ro consumption. We constructed a survey of Sen-Sei-Ro consumers with cancer to identify patients' demographic and medical characteristics, patterns of use, perceived benefits and rationale for use of this product. We also hoped to identify domains of interest for a patient-reported measure to be used in a controlled clinical trial of its effects on quality of life of cancer patients. Such an instrument, once validated, could also be applied more broadly to measuring the impact of other CAM products taken by cancer patients for purposes other than specific anti-tumor effects.

## Methods

A total of 2,346 Japanese consumers of Sen-Sei-Ro, listed in the Kyowa-S.S.I. database, were mailed a brief questionnaire in July 2001. Using a common industry practice of including questionnaires as product package inserts, the company had surveyed the consumption habits and health status of Sen-Sei-Ro consumers in 2000. Consumers who indicated they had cancer were eligible for the present survey. We developed a draft survey instrument in English, which was translated into Japanese and then back-translated into English to confirm the preservation of item definitions. A subsequent round of revisions was made. The instrument was mailed to current customers who in the previous survey had indicated that they either had been treated for cancer or used the product to mitigate the effects of cancer or cancer treatment.

The questionnaire included 37 items that assessed demographics (i.e., gender, age, and marital status), cancer history, consumption of Sen-Sei-Ro, and its perceived effects. Cancer status included questions about the primary tumor (i.e., 'Where did your cancer start?") and current treatment with intravenous chemotherapy, oral chemotherapy or radiation. It assessed consumption by asking when its use began relative to the cancer diagnosis and treatment and the amount and duration of use. Seven questions asked about the extent to which drinking Sen-Sei-Ro helps to: strengthen one's body so it can fight cancer and resist other illnesses, reduce symptoms of cancer and the side effects of treatment, cure cancer, feel better emotionally, and engage cancer spiritually. Finally, two items assessed motives for use: the importance of one's own efforts in fighting cancer, relative to "what doctors do," and family support for the notion that drinking it "will help me fight cancer."

Respondents' perceptions of more specific effects on quality of life were assessed by asking about 17 changes they may have noticed "about your body, how you feel physically, and how you feel emotionally" since they had been taking Sen-Sei-Ro. Response options included "better," "worse," and "about the same." These items were based on the previous survey, the clinical experiences of physicians in Korea who used it as part of their cancer patients' care [19], and our previous experience in assessing quality of life in cancer patients. The items were designed to assess a broad array of treatment side effects and aspects of physical function and emotional well being.

The analysis was designed to describe consumers' perceptions of the quality of life effects of using Sen-Sei-Ro and explore variation in their perceptions with respect to demographic and medical characteristics and product usage. In order to examine patterns in perceptions of the effects we conducted a principal components factor analysis with orthogonal rotation with the 17 individual effects items. Each item was coded so that increasing values indicated greater relief from symptoms. The factor results were then used to explore the definition of distinct, multi-item scales that could summarize perceptions of effects. Following psychometric convention, the Likert response set was considered and analyzed as an interval scale [20,21]. Such scales would offer increased overall reliability compared to reporting multiple individual items, as well as allow a more parsimonious description of the effects respondents attribute to using Sen-Sei-Ro. Associations between scale scores and ordered and nonordered categorical indicators of consumer characteristics and product usage were evaluated by calculating Spearman correlation coefficients, t-tests, and analyses of variance, as appropriate.

## Results

We received completed questionnaires from 782 (33%) of the sampled Sen-Sei-Ro consumers with a history of cancer. The respondents represented a broad range of cancer patients familiar with both cancer treatment and Sen-Sei-Ro. Slightly more than half were male (53%), 89% were married, and the median age was 65, with 87% between 51 and 80 years old (range: 31–91). Most respondents reported a single cancer, but 19% reported two or more (Table 1). Lung, colon and gastric cancer patients each accounted for one-fifth of diagnoses. However, patients with cancers that only or disproportionately affect women (uterus, cervix, and breast) outnumbered those with diagnoses confined to men (prostate), 191 (24%) vs. 75 (10%). Although most of these study participants were currently receiving cancer therapy, 37% were not, and 23% were receiving oral chemotherapy, radiation or both, and 39% were receiving intravenous chemotherapy, which is usually more toxic.

**Table 1:** Clinical Characteristics of 782 Consumers of Sen-Sei-Ro Who Responded to a Survey

**Characteristic**	**Number**	**Percent**
**Number of cancers reported**		
One	632	80.6
Two	129	16.5
Three or more	21	2.7
**Where cancer(s) started**		
Lung	177	23
Colon	170	22
Stomach	158	20
Liver	127	16
Breast	101	13
Ovaries	40	5
Cervix	29	4
Uterus	21	3
Prostate	75	10
Others (name of the organ or a part of body)	62	8
**Current treatment**		
No active treatment	292	37.4
Receiving oral chemotherapy or radiation only	181	23.2
Receiving intravenous chemotherapy?	307	39.4

Over 90% of the respondents had used the product for over 3 months, and over half for more than a year (Table 2). Only 3% of the study participants began using it before cancer was diagnosed, while approximately one-third initiated use after diagnosis, another third after treatment began and the remaining patients after treatment was completed. Nearly all used it daily, and 42% drank more than one pack each day.

**Table 2:** History of Use of Sen-Sei-Ro Reported by 782 Survey Respondents

Characteristic	Number	Percent
**How long have you been taking "Sen-Sei-Ro?**	(median: more than a year)	
Less than a month	9	1.2
1–3 months	57	7.3
3–6 months	108	13.9
6 months- 1 year	172	22.1
More than 1 year	432	55.5
**When did you start drinking "Sen-Sei-Ro?"**	(median: after starting treatment)	
Before the cancer diagnosis	22	2.9
At the time of cancer diagnosis (before surgery or chemotherapy)	218	28.4
After starting the treatment (surgery or chemotherapy)	266	34
After completion of treatment (surgery or chemotherapy)	262	34.1
**What best describes your situation?**	(median: every day)	
Drink it every day (routinely)	644	84.4
Drink while I am undergoing cancer treatment	71	9.3
Drink when I am feeling poorly, tired, and weak)	22	2.9
Drink when I am feeling worried about my cancer)	26	3.4
**How many packs do you drink, on the average, per week?**	(median: 1 pack/day)	
Less than 7 packs (less than 1/day)	49	6.4
7 packs (1 pack/day)	369	48.4
2 packs/day	195	25.6
3 packs/day	126	16.5
4 or more packs/day	24	3.1

These study participants expressed consistently positive views, though not extremely so, regarding general benefits. Between 62 and 74% said Sen-Sei-Ro helps in various respects, such as improving strength to fight cancer and ameliorating the side effects of treatment (Table 3). However, only 16% indicated it was a "great help" in building resistance to illnesses other than cancer, while 31% strongly endorsed its value in helping spiritually to fight cancer. The study participants endorsed two indicators of motivations for using it. Nearly all (93%) agreed, and a majority (51%) strongly, that relying on doctors alone is insufficient, implying they may believe that using Sen-Sei-Ro represents an important additional personal effort. Nearly all study participants (85%) also agreed that their families think it helps fight the cancer. Although the family's reported endorsement of this belief was somewhat weaker than the consumer's personal endorsement, virtually no one rejected it.

**Table 3:** Perceived Help from Sen-Sei-Ro by 782 Survey Respondents

	Percent		
**How much do you think that drinking "Sen-Sei-Ro" helps with the following?**	No help	Help	Great help

A. Improves my body strength to fight cancer	5	70	25
B. Improves my resistance against sickness other than cancer	10	74	16
C. Helps reduce symptoms of cancer	10	65	24
D. Helps reduce the side effects of cancer therapy	12	67	21
E. Helps with cancer treatment	10	67	24
F. Helps with improving emotional condition	9	66	25
G. Helps me spiritually to fight against cancer	6	62	31

### Positive and negative effects of Sen-Sei-Ro

The factor analysis of the 17 individual perceived effects items suggested two distinct constructs: Relief of Symptoms and Functional Well-being. The first referred to relief of specific symptoms and was defined by items pertaining to likely side effects of cancer and its treatment (e.g., appetite, weight loss, pain, nausea). The second factor was defined by perceptions of effects on strength, vitality, and emotional well-being. Subsequent evaluation of scales defined by these two sets of items, including item-scale discrimination and internal consistency, identified two items pertaining to effects on sleep and daytime drowsiness that discriminate poorly. That is, they were equally correlated with both scales, and thus were deleted. The remaining items displayed clear discrimination, correlating highly with their assigned scale and little with the other. As a result, we defined two conceptually and empirically distinct, and internally consistent (Cronbach's alpha: Relief of Symptoms, α = .74; Functional Well-Being, α = .91) summary scales of Sen-Sei-Ro study participants' perceptions of its effects relative to their cancer treatment. We present the useful items remaining after this analysis in Table 4. Scores were calculated by averaging the scores of the constituent items, and thus ranged from -1 to +1, with 0 indicating a perceived effect that was neither positive nor negative.

**Table 4:** Summary Scales of Noticed Effects of Using Sen-Sei-Ro From 782 Respondents

	**Treatment Symptoms**	**Functional Well-being**
	Item-Scale Correlations^1^	

Appetite	**0.50**	0.44
Maintained or gained weight	**0.53**	0.37
Lost weight	**0.48**	0.39
Feelings of pain	**0.54**	0.36
Ability to reduce hair loss or grow hair	**0.36**	0.28
Nausea or vomiting	**0.44**	0.38
Muscle weakness	0.39	**0.51**
Energy level	0.43	**0.75**
Physical strength	0.44	**0.73**
Feelings of tension, worry	0.38	**0.72**
Feelings of sadness, depression	0.43	**0.65**
Ability to spend time with other people	0.41	**0.70**
Ability to work or get chores done around the house	0.46	**0.75**
Overall sense of physical well being	0.50	**0.75**
Overall sense of emotional well being	0.39	**0.70**
Cronbach alpha	0.74	0.91

^1 ^Item-scale correlations, shown in bold face, are corrected of overlap.

Items included in factor analysis, but deleted from summary scales because of poor scale discrimination include: falling asleep quickly; feeling sleepy during the day;

Scale scores indicate that study participants reported generally favorable effects as indicated by the positive mean scores, although the majority of patients reported no effect either way on most individual items, and net scores of zero were reported by 26% and 37% for Functional Well-Being and Relief of Symptoms, respectively. The product was perceived to be somewhat more effective in promoting overall well-being (mean = .34, sd = .46) than in relieving symptoms (mean = .25, sd = .36). The most frequently reported specific benefits were for emotional (spiritual) well-being (50%), physical well-being (50%), energy level (44%), and strength (41%), with far fewer reporting benefit for specific muscle weakness. Large minorities (30% or more) felt it helped with anxiety or sadness, depression, daily activities and socializing with others. While Sen-Sei-Ro was less frequently seen as effective in alleviating common symptoms that are side effects of conventional treatment, a third or more reported benefits with respect to appetite and maintenance of body weight. More than 1 in 5 felt it helped with sleeplessness, pain and hair loss.

Perceived effects of using it were associated with gender, age, and tumor site. Women and those with uterine cancers reported better Functional Well-Being, but parallel trends for Relief of Symptoms did not achieve statistical significance (Table 5). Younger patients tended to report more positive effects on Functional Well-Being, but not on Relief of Symptoms. Surprisingly, current active anti-cancer treatment did not affect either scale, although patients who began using the product earlier in their course tended to report more benefit on Relief of Symptoms. The amount consumed each week was not associated with perceived effects in either domain (data not shown).

**Table 5:** Perceived Effects Scale Scores by Characteristics of 782 Respondents

		**Relief of Treatment Symptoms**	**test of association (p value)**	**Improve Functional Well-being**	**test of association (p value)**
		(mean scores)			

**All Respondents**		0.25		0.30	
Percent reporting no net effect		26%		37%	
**Sex**					
Male		0.23	t	0.25	t
Female		0.27	(0.092)	0.36	(< 0.001)
**Age group**					
20 – 49		0.30		0.42	
50 – 59		0.27	r = -0.03	0.29	r = -0.08
60 – 69		0.23	(0.389)	0.30	(0.016)
70 and older		0.25		0.26	
**Cancer group**					
Lung, stomach, liver		0.23	F	0.28	F
Colon, breast, uterus, cervix		0.29	(0.060)	0.35	(0.004)
Prostate, other		0.21		0.15	
Lung	No	0.27	t	0.32	t
	Yes	0.20	(0.032)	0.26	(0.190)
Stomach	No	0.25	t	0.29	t
	Yes	0.27	(0.379)	0.34	(0.245)
Liver	No	0.26	t	0.31	t
	Yes	0.18	(0.026)	0.23	(0.059)
Colon	No	0.24	t	0.29	t
	Yes	0.29	(0.074)	0.33	(0.295)
Breast	No	0.25	t	0.29	t
	Yes	0.26	(0.725)	0.36	(0.192)
Uterus	No	0.25	t	0.29	t
	Yes	0.39	(0.075)	0.50	(0.051)
Cervix	No	0.25	t	0.30	t
	Yes	0.26	(0.836)	0.42	(0.159)
Prostate	No	0.25	t	0.32	t
	Yes	0.21	(0.277)	0.14	(0.002)
**History using sen-sei-ro**					
≤ 3 months		0.14*		0.22	
3 – 6 months		0.24	r(s) = 0.07	0.30	r(s) = 0.04
6 – 12 months		0.25	(0.059)	0.29	(0.251)
> 12 months		0.27		0.32	
**Current treatment**					
No active treatment		0.24	F	0.31	F
Oral or radiation therapy		0.26	0.640	0.30	0.973
Intravenous chemotherapy		0.26		0.30	

t: t-test.

F: F, analysis of variance.

r: Pearson correlation coefficient; r(s): Spearman correlation coefficient.

* Group mean is significantly (p < 0.05) lower than means for other groups, which are not significantly different from one another

Study participants with aggressively treated, poor prognosis cancers (lung, gastric and liver) tended to report less benefit than those with less aggressively treated cancers (colon, breast, uterus or cervix). Those with prostate cancer and other cancers that seldom received chemotherapy during the survey period reported the least benefit.

## Discussion

This survey of consumers of Sen-Sei-Ro who reported a history of cancer found that nearly all began using it after their cancer was diagnosed, and those who started using it earlier in their cancer course tended to report greater benefit. Most patients report benefit from consuming this product and large proportions feel using it is a "great help." A survey of people currently using the product by choice produces a bias toward reported benefit, which our results reflect, and the relatively low 33% survey response rate probably increases the bias. However, despite the evident predisposition in favor of the product and the generally positive reports for more global benefits from its use, most of these study participants report little substantial effect in either direction when queried about specific consequences of cancer or its treatment.

Our findings shed some light on these patients and our measures. Our factor analysis of items focusing on 17 more or less specific effects indicated two major constructs that characterize study participants' perceptions of potential benefits of using Sen-Sei-Ro: relief of cancer and treatment-related symptoms and improvement in overall functional status and well-being, which fits with previous studies of cancer patients' motivations for pursuing CAM [4,22,23]. Like others, we found that improvements in functional well-being were greater than relief of symptoms, suggesting that cancer patients use CAM products for their effects, rather like a health promoting tonic, in strengthening their body's ability to recover from the debility of cancer treatment and support its ability to fight against cancer [22,24,25]. Our results provide some additional evidence of the validity of these measures. Younger patients, women, study participants with longer usage and thus more time to recover from initial cancer treatments, and those whose cancers had less poor prognoses and less aggressive treatment are all generally less symptomatic and in better overall health, and all tended to report more benefit by both measures. These results are consistent with both patients' limited understanding of pathophysiology and the sensitivity of these scales to their true condition: they felt better and thus able to ascribe benefit to Sen-Sei-Ro than others expected to be worse off. The still lower benefit reported by prostate cancer patients may reflect their better overall well-being and few symptoms from disease or treatment, providing little potential for benefit from Sen-Sei-Ro. The 2 scales postulated here appear sensitive to patient changes in two domains relevant to CAM use and may prove useful as outcome measures in future controlled trials of these substances in cancer patients.

Previous studies have helped to characterize who is likely to use dietary supplements, such as Sen-Sei-Ro and complementary and alternative medicine products more broadly. In studies of cancer patients and other populations in North America and Europe, the probability of choosing these approaches is greater with female gender, older age, higher education, lower body mass and indicators of healthy lifestyles, such as frequent exercise, avoidance of smoking, and diets low in fat and high in fruits and vegetables [26-32]. In a large national study in Japan, Ishihara et al. reported that 11% of men and 16% of women used dietary supplements, with use associated with older age, lower body mass, and frequent exercise, as well as longer work days (for men), and greater stress [32]. The reasons cancer patients give for using dietary supplements appear to revolve around three main themes, characterized in one study as surviving cancer (i.e., augmenting conventional treatment in fighting cancer), relieving both cancer symptoms and treatment side-effects, and repairing or boosting up, which includes detoxifying the body, boosting immunity and energy, and enhancing quality of life [22].

Other surveys of cancer patients have highlighted the goal of health restoration and promotion. The most common reasons for using CAM cited by breast cancer patients in Ontario were to boost the immune system (63%), increase quality of life (53%), and prevent cancer recurrence (43.5%) [4]. Large numbers of both women (73%) and men (56%) in the SEER cohort in western Washington used dietary supplements, primarily to improve general health and well being (95% of supplement users) [23]. However, relatively few cite symptom treatment as reason for using CAM: 21% mentioned treating side effects of conventional cancer treatments in the Ontario survey, and only 4% of the VA patients gave this reason. [4] A survey of cancer patients in the VA found that of the 61% who took a dietary supplement, 41% cited "energy increase" as their reason [24]. When asked about benefits, 40% said it improved overall health, 21% reported increased energy, and only 9% said dietary supplements improved symptoms. Yet, an MD Anderson survey found that having received chemotherapy doubled the likelihood of its use and was the most common characteristic of users [25]. Hedderson et al. found that cancer patients' decisions to use dietary supplements are associated with more severe treatment side effects and also a stronger desire for personal control [29].

The distinction between symptom relief and more global benefits echoes the distinction between the medical model of combating specific diseases and a more broadly understood relationship between diet and health. The concepts occasionally overlap, as in the well-established diet-heart theory linking dietary components, especially fats, to cardiac risk. However, the parallel diet-cancer theory has far less epidemiological support, and the concept of epidemic obesity in the US has apparently been embraced slowly and fitfully despite the striking data supporting it. The associations between CAM use and the healthy lifestyle and favorable socioeconomic variables, including higher educational and income levels, suggest that the broader concept of diet as a tonic, not a medicine, prevails in populations removed from severe economic adversity. As a result, the effects patients expect and perceive from Sen-Sei-Ro may have little to do with medical specifics such as the particular cancer diagnosis, the intensity of treatment and how recently it was given.

This distinction is useful for evaluating a substance taken both to obtain a global benefit, as well as to counter an array of specific symptoms. For example, antiemetic treatment against chemotherapy-induced nausea and vomiting produces quality of life benefits by reducing a specific noxious symptom complex that are much greater than the more global impact of erythropoietin on patients receiving chemotherapy. The two constructs we found correspond to patient goals for CAM identified in other studies. In another breast cancer patient cohort, CAM use was a marker of psychosocial distress, which may in part have motivated its use [33], since CAM is used more often by more seriously ill patients.

These results imply that patient-based measures of the effects CAM products must be strongly informed by patients' explanatory models of cancer "pharmacology," which may differ from their physicians', and measure the outcomes they value, according to their perspectives. Measures should focus on tonic effects on body strength and overall function as well as on symptom alleviation. If dietary supplements are not taken primarily for symptom relief, patients may not experience and report it, even if that is the reason their doctors prescribed the supplement. The stronger body patients hoped for may resist symptoms better, but the patient may consider that a lucky byproduct, not the main objective.

If the benefits of Sen-Sei-Ro and related products are likely to be global, rather than symptom-specific, investigations should target broad measures of well-being and systemic symptoms of anxiety or sadness, depression, appetite, maintaining body weight, daily activities, and socializing with others, in additional specific symptoms such as hair loss, nausea and pain. Our use of a 3-item Likert response set may be improved by using a 5-item scale, which would produce less granular data with better better statistical properties for psychometric analyses. We look forward to further development of this instrument to assess the use of this and other CAM substances in cancer patients. We plan to use these results to inform a series of focus groups to refine and supplement these items, pilot test additional items, subject the revised instrument to further validation studies and to use the final validated instrument in randomized trials of the efficacy of Sen-Sei-Ro and other CAM substances with putative health properties in cancer patients, if adequate research funding is available.

## Conclusion

Respondents to our survey of Sen-Sei-Ro consumers with cancer reported favorable perceived effects from its use. Our instrument, when further validated, may be a useful outcome in trials assessing this and other CAM substances in cancer patients.

## Competing interests

Drs. Talcott and Clark have consulted for and received research support from Sundory Corporation, which markets Sen-Sei-Ro. Dr. Lee is a Professor at the  Kanazawa University Venture Research Laboratory (VBL), to which Sundory and  Kyowa-S.S.I., Tokyo, Japan, which manufactures Sen-Sei-Ro, contribute research  funding.

## Authors' contributions

JAT was involved in the design of the study, the drafting of the survey, the interpretation of the results and drafted the manuscript. JAC was involved in the design of the study, the primary drafting of the survey, the execution and primary interpretation of the psychometric analyses and co-drafted the manuscript. IPL was involved in the concept and design of the study, coordinated the translation of the survey and the performance of the survey, and contributed to the draft and revisions of the manuscript. All authors read and approved the final manuscript.

## Pre-publication history

The pre-publication history for this paper can be accessed here:


